# 2,5-Dioxopyrrolidin-1-yl adamantane-1-carboxyl­ate

**DOI:** 10.1107/S1600536809024209

**Published:** 2009-07-04

**Authors:** Joe Liu, Jack K. Clegg, Rachel Codd

**Affiliations:** a School of Medical Sciences (Pharmacology) and Bosch Institute, D06, The University of Sydney, New South Wales 2006, Australia; b Centre for Heavy Metals Research, School of Chemistry, F11, University of Sydney, New South Wales 2006, Australia

## Abstract

The title compound, C_15_H_19_NO_4_, contains one crystallographically independent mol­ecule in the asymmetric unit. The N—O—C—O torsion angle is 1.97 (9)°. The two pairs of vicinal H atoms that lie above or below the plane defined by the five-membered pyrrolidine-2,5-dione ring are an average of 6.57 (5)° from being eclipsed. The average absolute C—C—C—C torsion angle in the adamantane skeleton, in which each fused cyclo­hexane ring is in a chair configuration, is 59.99 (5)°. The crystal packing is unremarkable.

## Related literature

For the biological activity of adamantane-1-carboxylic acid derivatives, see: De Felice *et al.* (2007 ▶); Jia *et al.* (2005 ▶); Stouffer *et al.* (2008 ▶). For related structures, see: Molčanov *et al.* (2006 ▶); Thackeray & White (1977 ▶); Homan *et al.* (1997 ▶). For related structures produced *via* biocatalysis, see: Bailey *et al.* (1996 ▶); Ridyard *et al.* (1996 ▶). For the structure of a derivative of the title compound, see the following paper: Liu *et al.* (2009 ▶).
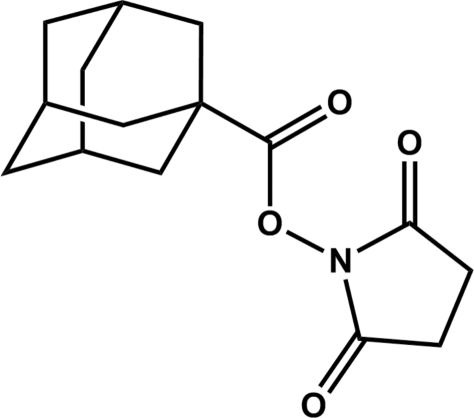


## Experimental

### 

#### Crystal data

C_15_H_19_NO_4_
*M* *_r_* = 277.31
Monoclinic, 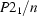

*a* = 6.6711 (3) Å
*b* = 29.4502 (14) Å
*c* = 7.0291 (3) Å
β = 104.447 (2)°
*V* = 1337.26 (10) Å^3^
*Z* = 4
Mo *K*α radiation
μ = 0.10 mm^−1^
*T* = 150 K
0.30 × 0.28 × 0.10 mm

#### Data collection

Bruker APEXII–FR591 diffractometer
Absorption correction: multi-scan (*SADABS*; Sheldrick, 2007 ▶) *T*_min_ = 0.888, *T*_max_ = 0.990
51912 measured reflections
6819 independent reflections
6104 reflections with *I* > 2σ(*I*)
*R*_int_ = 0.032

#### Refinement

*R*[*F* ^2^ > 2σ(*F* ^2^)] = 0.042
*wR*(*F* ^2^) = 0.120
*S* = 1.08
6819 reflections
181 parameters
H-atom parameters constrained
Δρ_max_ = 0.44 e Å^−3^
Δρ_min_ = −0.29 e Å^−3^

### 

Data collection: *APEX2* (Bruker, 2003 ▶); cell refinement: *SAINT* (Bruker, 2003 ▶); data reduction: *SAINT* and *XPREP* (Bruker, 2003 ▶); program(s) used to solve structure: *SIR97* (Altomare *et al.*, 1999 ▶); program(s) used to refine structure: *SHELXL97* (Sheldrick, 2008 ▶); molecular graphics: *ORTEP-3* (Farrugia, 1997 ▶), *WinGX32* (Farrugia, 1999 ▶) and *POV-RAY* (Cason, 2002 ▶); software used to prepare material for publication: *enCIFer* (Allen *et al.*, 2004 ▶).

## Supplementary Material

Crystal structure: contains datablocks global, I. DOI: 10.1107/S1600536809024209/bg2265sup1.cif

Structure factors: contains datablocks I. DOI: 10.1107/S1600536809024209/bg2265Isup2.hkl

Additional supplementary materials:  crystallographic information; 3D view; checkCIF report

## Figures and Tables

**Fig. 1.**
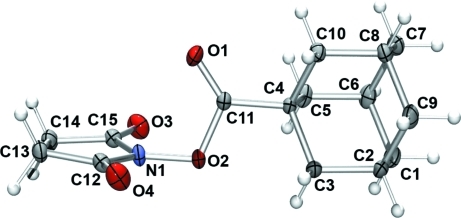
ORTEP representation of I shown with 50% probability ellipsoids.

## supplementary crystallographic information

### Comment

Adamantane-1-carboxylate-2,5-pyrrolidinedione (I) (Fig. 1.) was prepared in our laboratory as part of our bioconjugate program in drug design. Adamantane-1-carboxylic acid belongs to a family of functionalized polycyclic cage-based compounds that have relevance in the design of therapeutics, with several compounds in clinical use for the treatment of influenza (amantadine) (Stouffer *et al.*, 2008), Alzheimer's disease (memantine) (De Felice *et al.*, 2007) and pulmonary tuberculosis (SQ109) (Jia *et al.*, 2005). The torsional bond in I defined by atoms N1—O2—C11—O1 is 1.97 (9) °. The distance between the 2,5-pyrrolidinedione-derived oxo groups and the carbonyl O atom in I (O1) is 3.52 (1) Å (O4–O1) or 3.39 (1) Å (O3—O1); this difference arises from the O4 group lying 0.10 (1) Å below the plane defined by N1, C13 and C14 and the O3 group lying 0.28 (1) Å above this same plane and on the same side as O1. Amide conjugates of adamantane-1-carboxylic acid might furnish compounds with the ability to traverse cell membranes.

### Experimental

A white precipitate of I was formed after the addition of water (1 ml) to a cooled solution of DMF (10 ml) containing *N*-hydroxysuccinimide (NHS: 0.29 g, 2.55 mmol), 1-ethyl-3-(3-dimethylaminopropyl)carbodiimide (EDC: 0.39 g, 2.00 mmol) and adamantane-1-carboxylic acid (0.46 g, 2.55 mmol) that had been heated to 40 ° C for 4 h. The product was dried *in vacuo*; colourless crystals of I appeared after approximately 1 month from a 4.5 mg m*L*^-1^ solution of I in ethanol:water (7:3).

### Refinement

C and N bound-H (atoms were included in idealized positions and refined using a riding-model approximation, with C—H bond lengths fixed at 1.00 Å, 0.99 Å,for methine and methylene H atoms respectively. *U*_iso_(H) values were fixed at 1.2*U*_eq_ of the parent atoms for all H atoms.

### Crystal data

**Table:**

C_15_H_19_NO_4_	*F*(000) = 592
*M**_r_* = 277.31	*D*_x_ = 1.377 Mg m^−^^3^
Monoclinic, *P*2_1_/*n*	Mo *K*α radiation, λ = 0.71073 Å
Hall symbol: -P 2yn	Cell parameters from 9946 reflections
*a* = 6.6711 (3) Å	θ = 2.8–37.1°
*b* = 29.4502 (14) Å	µ = 0.10 mm^−^^1^
*c* = 7.0291 (3) Å	*T* = 150 K
β = 104.447 (2)°	Plate, colourless
*V* = 1337.26 (10) Å^3^	0.30 × 0.28 × 0.10 mm
*Z* = 4	

### Data collection

**Table:**

Bruker APEXII–FR591 diffractometer	6819 independent reflections
Radiation source: rotating anode	6104 reflections with *I* > 2σ(*I*)
graphite	*R*_int_ = 0.032
ω+φ scans	θ_max_ = 37.2°, θ_min_ = 2.8°
Absorption correction: multi-scan (*SADABS*; Sheldrick, 2007)	*h* = −11→11
*T*_min_ = 0.888, *T*_max_ = 0.990	*k* = −49→50
51912 measured reflections	*l* = −11→11

### Refinement

**Table:**

Refinement on *F*^2^	Primary atom site location: structure-invariant direct methods
Least-squares matrix: full	Secondary atom site location: difference Fourier map
*R*[*F*^2^ > 2σ(*F*^2^)] = 0.042	Hydrogen site location: inferred from neighbouring sites
*wR*(*F*^2^) = 0.120	H-atom parameters constrained
*S* = 1.08	*w* = 1/[σ^2^(*F*_o_^2^) + (0.057*P*)^2^ + 0.2898*P*] where *P* = (*F*_o_^2^ + 2*F*_c_^2^)/3
6819 reflections	(Δ/σ)_max_ < 0.001
181 parameters	Δρ_max_ = 0.44 e Å^−^^3^
0 restraints	Δρ_min_ = −0.29 e Å^−^^3^

### Special details

**Table:**

Experimental. The crystal was coated in Exxon Paratone N hydrocarbon oil and mounted on a thin mohair fibre attached to a copper pin. Upon mounting on the diffractometer, the crystal was quenched to 150(K) under a cold nitrogen gas stream supplied by an Oxford Cryosystems Cryostream and data were collected at this temperature.
Geometry. All e.s.d.'s (except the e.s.d. in the dihedral angle between two l.s. planes) are estimated using the full covariance matrix. The cell e.s.d.'s are taken into account individually in the estimation of e.s.d.'s in distances, angles and torsion angles; correlations between e.s.d.'s in cell parameters are only used when they are defined by crystal symmetry. An approximate (isotropic) treatment of cell e.s.d.'s is used for estimating e.s.d.'s involving l.s. planes.
Refinement. Refinement of *F*^2^ against ALL reflections. The weighted *R*-factor *wR* and goodness of fit *S* are based on *F*^2^, conventional *R*-factors *R* are based on *F*, with *F* set to zero for negative *F*^2^. The threshold expression of *F*^2^ > σ(*F*^2^) is used only for calculating *R*-factors(gt) *etc*. and is not relevant to the choice of reflections for refinement. *R*-factors based on *F*^2^ are statistically about twice as large as those based on *F*, and *R*- factors based on ALL data will be even larger.

### Fractional atomic coordinates and isotropic or equivalent isotropic displacement parameters (Å^2^)

**Table:**

	*x*	*y*	*z*	*U*_iso_*/*U*_eq_	
C1	1.22052 (11)	0.15040 (3)	0.40895 (12)	0.02581 (14)	
H1A	1.2692	0.1204	0.3753	0.031*	
H1B	1.3395	0.1666	0.4946	0.031*	
C2	1.04957 (11)	0.14401 (2)	0.51785 (10)	0.02258 (13)	
H2	1.1055	0.1262	0.6407	0.027*	
C3	0.86612 (11)	0.11819 (2)	0.38550 (10)	0.02168 (12)	
H3A	0.7567	0.1135	0.4562	0.026*	
H3B	0.9127	0.0881	0.3509	0.026*	
C4	0.77940 (9)	0.146072 (19)	0.19724 (8)	0.01413 (9)	
C5	0.95139 (10)	0.15250 (3)	0.08694 (9)	0.02086 (11)	
H5A	0.9979	0.1225	0.0507	0.025*	
H5B	0.8966	0.1700	−0.0352	0.025*	
C6	1.13503 (10)	0.17802 (3)	0.22017 (11)	0.02310 (12)	
H6	1.2464	0.1821	0.1491	0.028*	
C7	1.06322 (11)	0.22477 (2)	0.27366 (11)	0.02349 (13)	
H7A	1.1817	0.2414	0.3574	0.028*	
H7B	1.0089	0.2427	0.1527	0.028*	
C8	0.89390 (11)	0.21863 (2)	0.38360 (10)	0.01981 (11)	
H8	0.8472	0.2491	0.4188	0.024*	
C9	0.97680 (12)	0.19081 (3)	0.57139 (10)	0.02357 (13)	
H9A	0.8665	0.1869	0.6417	0.028*	
H9B	1.0939	0.2071	0.6593	0.028*	
C10	0.70934 (10)	0.19322 (2)	0.25152 (10)	0.01816 (10)	
H10A	0.6530	0.2110	0.1304	0.022*	
H10B	0.5985	0.1897	0.3214	0.022*	
C11	0.59920 (9)	0.12338 (2)	0.05527 (9)	0.01710 (10)	
C12	0.27538 (10)	0.04257 (2)	−0.03135 (10)	0.01826 (10)	
C13	0.14462 (10)	0.02270 (2)	−0.22012 (10)	0.02094 (11)	
H13A	0.0197	0.0415	−0.2712	0.025*	
H13B	0.1006	−0.0086	−0.1981	0.025*	
C14	0.28296 (11)	0.02249 (2)	−0.36545 (10)	0.02184 (12)	
H14A	0.3150	−0.0090	−0.3971	0.026*	
H14B	0.2134	0.0382	−0.4888	0.026*	
C15	0.47840 (10)	0.04733 (2)	−0.26279 (10)	0.01859 (11)	
N1	0.46311 (9)	0.054259 (19)	−0.07064 (8)	0.01894 (10)	
O1	0.45874 (9)	0.14099 (2)	−0.06059 (9)	0.02874 (13)	
O2	0.61824 (8)	0.075815 (16)	0.06775 (8)	0.02138 (10)	
O3	0.62486 (10)	0.05906 (2)	−0.32307 (10)	0.02936 (12)	
O4	0.23579 (11)	0.04800 (2)	0.12597 (9)	0.02915 (12)	

### Atomic displacement parameters (Å^2^)

**Table:**

	*U*^11^	*U*^22^	*U*^33^	*U*^12^	*U*^13^	*U*^23^
C1	0.0157 (3)	0.0261 (3)	0.0305 (3)	0.0019 (2)	−0.0038 (2)	−0.0024 (2)
C2	0.0245 (3)	0.0195 (2)	0.0178 (2)	−0.0049 (2)	−0.0058 (2)	0.00400 (19)
C3	0.0244 (3)	0.0172 (2)	0.0185 (2)	−0.0061 (2)	−0.0039 (2)	0.00501 (18)
C4	0.0131 (2)	0.01371 (19)	0.01396 (19)	−0.00133 (16)	0.00043 (16)	0.00029 (15)
C5	0.0171 (2)	0.0291 (3)	0.0169 (2)	−0.0011 (2)	0.00514 (19)	−0.0038 (2)
C6	0.0144 (2)	0.0322 (3)	0.0230 (3)	−0.0042 (2)	0.0052 (2)	−0.0018 (2)
C7	0.0217 (3)	0.0217 (3)	0.0246 (3)	−0.0085 (2)	0.0012 (2)	0.0042 (2)
C8	0.0196 (3)	0.0159 (2)	0.0218 (3)	−0.00142 (18)	0.0012 (2)	−0.00345 (18)
C9	0.0275 (3)	0.0261 (3)	0.0153 (2)	−0.0063 (2)	0.0020 (2)	−0.0037 (2)
C10	0.0148 (2)	0.0168 (2)	0.0216 (2)	0.00051 (17)	0.00218 (19)	−0.00262 (18)
C11	0.0156 (2)	0.0163 (2)	0.0173 (2)	−0.00132 (17)	0.00011 (18)	−0.00125 (17)
C12	0.0194 (2)	0.0146 (2)	0.0210 (2)	−0.00237 (18)	0.0057 (2)	−0.00127 (18)
C13	0.0168 (2)	0.0197 (2)	0.0248 (3)	−0.00409 (19)	0.0022 (2)	−0.0032 (2)
C14	0.0234 (3)	0.0216 (3)	0.0189 (2)	−0.0042 (2)	0.0023 (2)	−0.0043 (2)
C15	0.0193 (2)	0.0166 (2)	0.0199 (2)	−0.00113 (18)	0.0051 (2)	−0.00084 (18)
N1	0.0178 (2)	0.0196 (2)	0.0182 (2)	−0.00664 (17)	0.00223 (17)	−0.00448 (16)
O1	0.0241 (3)	0.0233 (2)	0.0296 (3)	0.00305 (18)	−0.0106 (2)	−0.00286 (19)
O2	0.0205 (2)	0.01610 (18)	0.0224 (2)	−0.00444 (15)	−0.00436 (17)	−0.00179 (15)
O3	0.0271 (3)	0.0312 (3)	0.0341 (3)	−0.0051 (2)	0.0157 (2)	−0.0009 (2)
O4	0.0369 (3)	0.0291 (3)	0.0254 (2)	−0.0057 (2)	0.0152 (2)	−0.0041 (2)

### Geometric parameters (Å, °)

**Table:**

C1—C2	1.5354 (12)	C8—C9	1.5348 (10)
C1—C6	1.5395 (11)	C8—C10	1.5386 (9)
C1—H1A	0.9900	C8—H8	1.0000
C1—H1B	0.9900	C9—H9A	0.9900
C2—C9	1.5387 (11)	C9—H9B	0.9900
C2—C3	1.5406 (9)	C10—H10A	0.9900
C2—H2	1.0000	C10—H10B	0.9900
C3—C4	1.5421 (8)	C11—O1	1.1956 (8)
C3—H3A	0.9900	C11—O2	1.4072 (8)
C3—H3B	0.9900	C12—O4	1.2099 (9)
C4—C11	1.5130 (8)	C12—N1	1.3913 (9)
C4—C10	1.5431 (8)	C12—C13	1.5121 (9)
C4—C5	1.5483 (9)	C13—C14	1.5381 (10)
C5—C6	1.5394 (10)	C13—H13A	0.9900
C5—H5A	0.9900	C13—H13B	0.9900
C5—H5B	0.9900	C14—C15	1.5120 (9)
C6—C7	1.5351 (11)	C14—H14A	0.9900
C6—H6	1.0000	C14—H14B	0.9900
C7—C8	1.5301 (11)	C15—O3	1.2083 (9)
C7—H7A	0.9900	C15—N1	1.3946 (9)
C7—H7B	0.9900	N1—O2	1.3849 (7)
			
C2—C1—C6	109.45 (5)	C7—C8—C10	109.46 (5)
C2—C1—H1A	109.8	C9—C8—C10	108.77 (5)
C6—C1—H1A	109.8	C7—C8—H8	109.5
C2—C1—H1B	109.8	C9—C8—H8	109.5
C6—C1—H1B	109.8	C10—C8—H8	109.5
H1A—C1—H1B	108.2	C8—C9—C2	109.62 (5)
C1—C2—C9	109.33 (6)	C8—C9—H9A	109.7
C1—C2—C3	109.66 (6)	C2—C9—H9A	109.7
C9—C2—C3	109.77 (6)	C8—C9—H9B	109.7
C1—C2—H2	109.4	C2—C9—H9B	109.7
C9—C2—H2	109.4	H9A—C9—H9B	108.2
C3—C2—H2	109.4	C8—C10—C4	109.92 (5)
C2—C3—C4	109.07 (5)	C8—C10—H10A	109.7
C2—C3—H3A	109.9	C4—C10—H10A	109.7
C4—C3—H3A	109.9	C8—C10—H10B	109.7
C2—C3—H3B	109.9	C4—C10—H10B	109.7
C4—C3—H3B	109.9	H10A—C10—H10B	108.2
H3A—C3—H3B	108.3	O1—C11—O2	121.17 (6)
C11—C4—C3	113.39 (5)	O1—C11—C4	128.04 (6)
C11—C4—C10	108.71 (5)	O2—C11—C4	110.74 (5)
C3—C4—C10	109.81 (5)	O4—C12—N1	124.15 (6)
C11—C4—C5	106.85 (5)	O4—C12—C13	130.05 (7)
C3—C4—C5	109.19 (5)	N1—C12—C13	105.80 (5)
C10—C4—C5	108.77 (5)	C12—C13—C14	105.90 (5)
C6—C5—C4	109.37 (5)	C12—C13—H13A	110.6
C6—C5—H5A	109.8	C14—C13—H13A	110.6
C4—C5—H5A	109.8	C12—C13—H13B	110.6
C6—C5—H5B	109.8	C14—C13—H13B	110.6
C4—C5—H5B	109.8	H13A—C13—H13B	108.7
H5A—C5—H5B	108.2	C15—C14—C13	105.66 (5)
C7—C6—C5	109.70 (6)	C15—C14—H14A	110.6
C7—C6—C1	109.50 (6)	C13—C14—H14A	110.6
C5—C6—C1	109.51 (6)	C15—C14—H14B	110.6
C7—C6—H6	109.4	C13—C14—H14B	110.6
C5—C6—H6	109.4	H14A—C14—H14B	108.7
C1—C6—H6	109.4	O3—C15—N1	123.96 (6)
C8—C7—C6	109.43 (5)	O3—C15—C14	130.34 (7)
C8—C7—H7A	109.8	N1—C15—C14	105.68 (5)
C6—C7—H7A	109.8	O2—N1—C12	121.72 (5)
C8—C7—H7B	109.8	O2—N1—C15	121.67 (6)
C6—C7—H7B	109.8	C12—N1—C15	116.30 (5)
H7A—C7—H7B	108.2	N1—O2—C11	111.87 (5)
C7—C8—C9	110.14 (6)		
			
C6—C1—C2—C9	−60.05 (7)	C3—C4—C10—C8	59.67 (7)
C6—C1—C2—C3	60.35 (7)	C5—C4—C10—C8	−59.76 (6)
C1—C2—C3—C4	−60.62 (7)	C3—C4—C11—O1	151.19 (8)
C9—C2—C3—C4	59.51 (8)	C10—C4—C11—O1	28.75 (9)
C2—C3—C4—C11	179.35 (6)	C5—C4—C11—O1	−88.47 (9)
C2—C3—C4—C10	−58.83 (7)	C3—C4—C11—O2	−31.38 (8)
C2—C3—C4—C5	60.35 (7)	C10—C4—C11—O2	−153.82 (5)
C11—C4—C5—C6	176.77 (5)	C5—C4—C11—O2	88.96 (6)
C3—C4—C5—C6	−60.23 (7)	O4—C12—C13—C14	−177.07 (7)
C10—C4—C5—C6	59.58 (7)	N1—C12—C13—C14	2.61 (7)
C4—C5—C6—C7	−60.28 (7)	C12—C13—C14—C15	−6.58 (7)
C4—C5—C6—C1	59.93 (7)	C13—C14—C15—O3	−173.15 (7)
C2—C1—C6—C7	60.38 (7)	C13—C14—C15—N1	8.13 (7)
C2—C1—C6—C5	−59.95 (8)	O4—C12—N1—O2	−3.72 (10)
C5—C6—C7—C8	60.42 (7)	C13—C12—N1—O2	176.58 (5)
C1—C6—C7—C8	−59.79 (7)	O4—C12—N1—C15	−177.39 (7)
C6—C7—C8—C9	59.43 (7)	C13—C12—N1—C15	2.91 (8)
C6—C7—C8—C10	−60.11 (7)	O3—C15—N1—O2	0.32 (10)
C7—C8—C9—C2	−59.33 (7)	C14—C15—N1—O2	179.14 (6)
C10—C8—C9—C2	60.63 (7)	O3—C15—N1—C12	173.99 (7)
C1—C2—C9—C8	59.41 (7)	C14—C15—N1—C12	−7.19 (8)
C3—C2—C9—C8	−60.91 (8)	C12—N1—O2—C11	−89.36 (7)
C7—C8—C10—C4	60.29 (7)	C15—N1—O2—C11	83.97 (7)
C9—C8—C10—C4	−60.09 (7)	O1—C11—O2—N1	1.97 (9)
C11—C4—C10—C8	−175.76 (5)	C4—C11—O2—N1	−175.66 (5)
